# CAML Does Not Modulate Tetherin-Mediated Restriction of HIV-1 Particle Release

**DOI:** 10.1371/journal.pone.0009005

**Published:** 2010-02-02

**Authors:** Mohammed S. Ali, Jason Hammonds, Lingmei Ding, Paul Spearman

**Affiliations:** Department of Pediatrics, Emory University and Children's Healthcare of Atlanta, Atlanta, Georgia, United States of America; University of California San Francisco, United States of America

## Abstract

**Background:**

Tetherin/BST-2 is a recently-identified potent restriction factor in human cells that restricts HIV particle release following particle formation and budding at the plasma membrane. Vpu counteracts tetherin's restriction of particle release in a manner that has not yet been fully defined. We recently identified calcium-modulating cyclophilin ligand (CAML) as a Vpu-interacting protein that also restricts particle release. We hypothesized that CAML may act to enhance tetherin-mediated restriction of particle release and thereby explain how two distinct factors could be responsible for Vpu-responsive restriction.

**Methodology/Principal Findings:**

Endogenous levels of tetherin in human cells correlated well with their restriction pattern and responsiveness to Vpu, while levels of cellular CAML protein did not. Tetherin but not CAML was inducible by interferon in a wide variety of human cells. Stable depletion of human CAML in restrictive HeLa cells had no effect on cell surface levels of tetherin, and failed to relieve tetherin-mediated restriction. Stable depletion of tetherin from HeLa cells, in contrast, rendered HeLa cells permissive and Vpu-unresponsive. Tetherin but not CAML expression in permissive human cells rendered them restrictive and Vpu responsive. Depletion of CAML had no influence on cell surface levels of tetherin.

**Conclusions/Significance:**

We conclude that tetherin restricts particle release and does not require CAML for this effect. Furthermore, these results do not support a major role for CAML in restricting HIV particle release in human cells.

## Introduction

Vpu is an 81-amino acid protein that is translated from a bicistronic mRNA which also encodes the envelope glycoprotein [1], [2]. Vpu has two known functions that appear distinct [3]. One of the well-described roles for Vpu is in degradation of CD4 through the formation of a ternary complex consisting of Vpu, CD4, and βTrCP [4], [5], [6], [7]. A second function of Vpu that was recognized in early studies and is now receiving increased attention is a role in enhancing particle release [8], [9], [10]. Heterokaryon studies between restrictive, Vpu-responsive human cells and permissive, Vpu-unresponsive simian cells led to the concept that Vpu enhances release by overcoming a dominant host restriction [11]. The restriction to particle release was subsequently shown to enhance endocytosis of retained particles, and to inducible by interferon alpha [9], [12]. In the past year, two distinct molecules have been identified as human host cell restriction factors that are counteracted by Vpu. Tetherin (also known as BST-2) was identified by the Bieniasz and Guatelli laboratories [13], [14] and calcium-modulating cyclophilin ligand (CAML) by our laboratory [15].

Bone marrow stromal cell surface gene (BST-2) was described originally as a novel human membrane protein cloned from a synovial cell line that was thought to be involved in pre-B cell growth [16]. A surface antigen overexpressed on multiple myeloma cells known at HM1.24 was subsequently shown to be identical to BST-2 [17]. BST-2 is an unusual type II membrane protein that is connected to the membrane via its N-terminal transmembrane portion and via a C-terminal GPI anchor [18]. Using a membrane proteomics approach, Bartee and coworkers found that BST-2 was downmodulated by the KSHV K5 protein, a RING-type E3 ubiquitin ligase known to be an immune modulator [19]. BST-2 was renamed tetherin by the Bieniasz laboratory when it was discovered that this molecule is involved in tethering of HIV particles at the plasma membrane [13]. These investigators found that tetherin can convey resistance to particle release when expressed in permissive cells, and that depletion of tetherin from restrictive human cells relieved the restriction. Most importantly, the restriction was specifically relieved by Vpu. The Guatelli group subsequently demonstrated that Vpu expression downmodulates tetherin/BST-2 from the cell surface [14]. Thus tetherin fits very well as a new host restriction factor that acts at the level of particle release and is overcome by Vpu.

CAML is a ubiquitous protein that was originally identified as a cyclophilin B-binding protein and plays an important role in T cell signaling [20], [21]. CAML is an ER-resident, type II integral membrane protein with three putative transmembrane domains at its C-terminus. CAML expression induces calcium-mediated signaling in T lymphocytes [22], and is required for efficient recycling of EGF receptor [23] and of GABA_A_ receptors [24] to the cell surface. Our group identified CAML as a Vpu-interacting protein through a yeast 2-hybrid approach, and expression and depletion studies revealed that CAML shared many of the same characteristics of a host restriction factor acting at the stage of particle retention [15]. Expression of Vpu or of the HIV-2 envelope glycoprotein counteracted the restriction posed by CAML. We therefore proposed that CAML either acts as an independent restriction factor at the same stage of replication as tetherin, or that it might modulate the restriction posed by tetherin. One attractive model that could tie both factors together would be a role for CAML in the recycling of tetherin to the cell surface, similar to the role of CAML in the recycling of the EGF receptor [23].

This study sought to define the role of CAML in tetherin-mediated restriction of HIV particle release. We reproduced findings from the Bieniasz laboratory demonstrating the potent Vpu-responsive restriction of particle release conferred by tetherin. Stable cell lines with depletion of CAML or tetherin were created to probe the dependence of one factor on the other. No effects of CAML depletion on cell surface tetherin were demonstrable, while Vpu expression led to downmodulation of cell surface tetherin. Stable depletion of tetherin relieved the restriction to particle release in HeLa cells, while stable depletion of CAML had no effect. These results, combined with the correlation of endogenous tetherin levels and restrictive cell phenotype, strongly support the role of tetherin as a potent, Vpu-responsive restriction factor and indicate that CAML is not required for restriction of particle release.

## Results

### Direct Comparison of Endogenous CAML and Tetherin Levels in Permissive and Restrictive Cells

We first sought to understand more completely the relationship between endogenous protein levels and restrictive/permissive cell phenotype, as this should provide a powerful indication of the role of CAML and tetherin in contributing to these phenotypes. To facilitate this study, we developed specific rabbit antisera to the cytoplasmic domain of CAML and the ectodomain of tetherin. We examined the level of CAML and tetherin RNA and protein levels in permissive and restrictive cells. To add to the utility of this comparison, we performed the analysis on the same cell types following stimulation with interferon alpha. Three permissive human cell types (293T, HOS, and HT1080) and three restrictive cell types (HeLa, A3.01 T cell line, and Jurkat T cell line) were examined, along with the permissive African green monkey cell line Cos-7.

**Figure 1:**
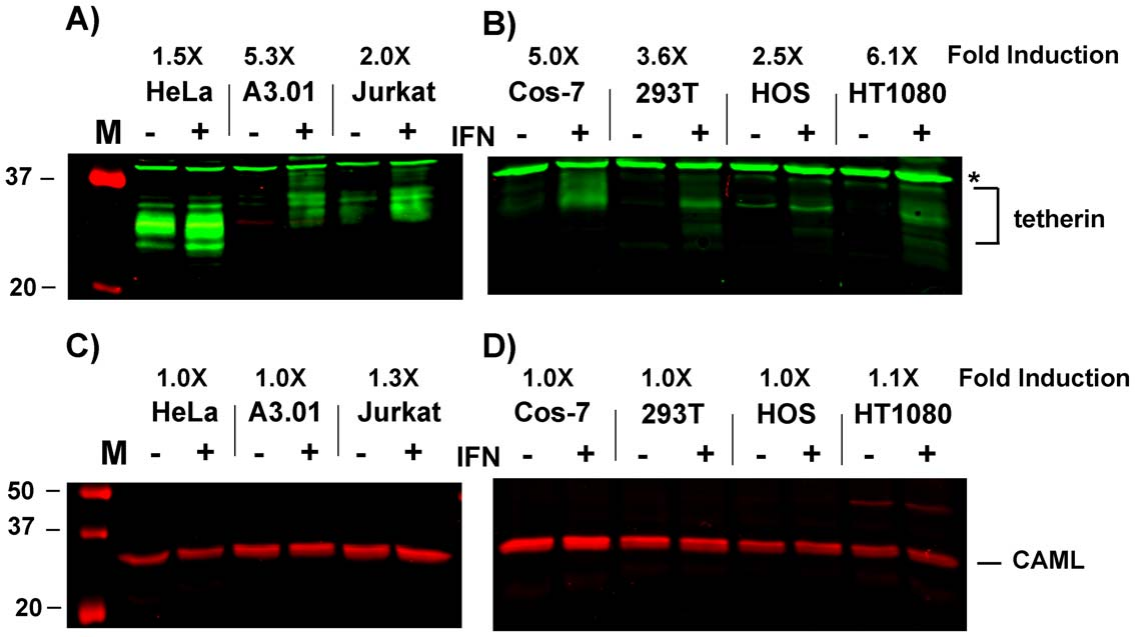
Tetherin is interferon-inducible; CAML is not interferon-inducible. A) Western blot for endogenous tetherin was performed using rabbit polyclonal antisera before or 48 hours after interferon-induction. Note that induction was demonstrated in each case, and baseline levels of tetherin were highest in HeLa cells. Lanes were normalized by total protein quantitation. D) Western blotting for endogenous CAML was performed in the same panel of cells. Note that no induction was demonstrated, and all cell types expressed detectable CAML. Cell lines are indicated above the blots, and IFN treatment is indicated with a plus sign. M  =  molecular mass markers.

To confirm the interferon-inducible nature of tetherin and investigate further the inducibility of CAML, we performed Western blots on samples from each of the cell lines, normalizing for total protein loaded. Using tetherin-specific polyclonal antisera, a series of bands representing variably-glycosylated tetherin monomers were noted of 30–34 Kd molecular mass, and in some samples a slower migrating series of bands were noted (Fig. 1A). HeLa cells demonstrated substantial levels of tetherin in the uninduced samples. Jurkat cells had visibly less tetherin in the unstimulated samples, and it was difficult to appreciate tetherin bands in A3.01 cells until after interferon treatment (Fig. 1A). Note that the antisera detected a non-specific cross-reacting bands (asterisks). Nevertheless, the intensity of tetherin detected in each of the restrictive cell types increased markedly following interferon alpha treatment. The detection of tetherin protein was somewhat more difficult in permissive cells, and very little tetherin was seen in any of the uninduced permissive cells. Following interferon treatment, substantial increases in tetherin were noted in all four cell lines examined, including Cos-7 cells (Fig. 1B). The enhancement of tetherin expression in HOS cells was less prominent than that seen in HT1080 cells or Cos-7 cells. Induction varied from 1.5-fold for HeLa to a maximum of 6.1-fold for HT1080 (Fig. 1A and 1B). These results confirm the inducibility of tetherin by interferon alpha at the protein level, and overall support a model in which tetherin is an interferon-inducible host restriction factor. We then examined CAML protein levels in the same panel of cells by Western blot. CAML was easily detected in all restrictive and permissive cells (Fig. 1C and 1D). There was some variation in protein level between cell lines, but this minor variation did not correlate with segregation of cells into restrictive, Vpu-responsive phenotype or permissive, Vpu-unresponsive categories. Notably, CAML levels in HeLa cells were not significantly different than 293T or HOS, and permissive Cos-7 cells demonstrated a significant amount of CAML. There was a near complete absence of response to interferon in any of the cell lines (Fig. 1C and 1D, fold-induction shown above). These results demonstrate that CAML is not inducible by alpha interferon, and there is not an apparent correlation between CAML levels and the restrictive phenotype for particle release at either the RNA or protein level.

### CAML Is Not Required for Tetherin-Mediated Restriction

**Figure 2:**
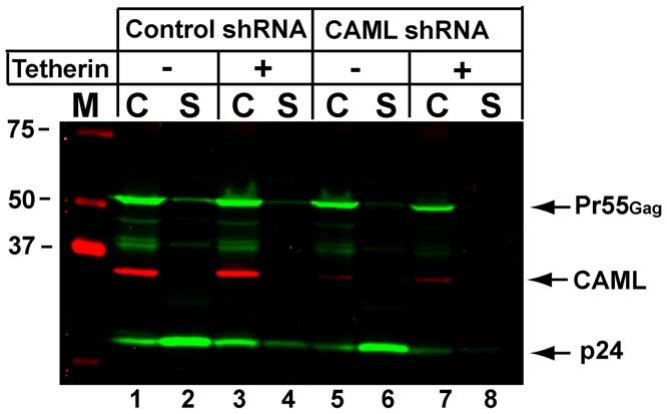
CAML depletion does not inhibit tetherin-mediated restriction of particle release. 293T cells were transduced with an shRNA-encoding lentivirus expressing control shRNA or CAML shRNA and selected with puromycin. Selected cell populations were then transfected with tetherin expression vector and NLUdel proviral DNA. Cells (C) and supernatants (S) harvested at 48 hours for Western blot analysis. Particles present in supernatants were pelleted through 20% sucrose prior to loading. Analysis was performed using infrared detection, allowing simultaneous assessment of CAML in cell lysates by rabbit polyclonal antisera (red) and HIV Gag proteins by anti-p24 monoclonal antibody (green). Co-transfection of cells with a tetherin expression construct is indicated by plus signs. M  =  molecular mass markers.

Although CAML protein levels appeared unrelated to restrictive cell phenotype, we hypothesized that CAML might play an important accessory role in tetherin-mediated restriction of particle release. To address this possibility, we first utilized the permissive 293T cell line. Employing lentivirus-mediated shRNA transduction, we generated 293T cells that were depleted of endogenous CAML. CAML was depleted by over 90% in these cells (red band at 34 Kd, Fig. 2, compare lanes 1 and 3 with lanes 5 and 7). Control shRNA-transduced 293T cells were employed in parallel (lanes 1–4). Cells were transfected with pNL4-3 bearing an in-frame deletion of the vpu ORF (NLUdel, [8]) in the presence or absence of expression of tetherin, and particle output monitored by Western blotting. In control cells with normal levels of CAML, the expression of tetherin resulted in a marked restriction of particle release as indicated by p24 in the supernatant (Fig. 2, compare lanes 2 and 4). In cells with CAML depletion, there was no difference in particle output from control cells (compare lane 6 to lane 2), and tetherin expression still induced a potent restriction to particle release (lane 8). These results suggested that CAML levels do not modulate tetherin-mediated restriction in 293T cells.

**Figure 3:**
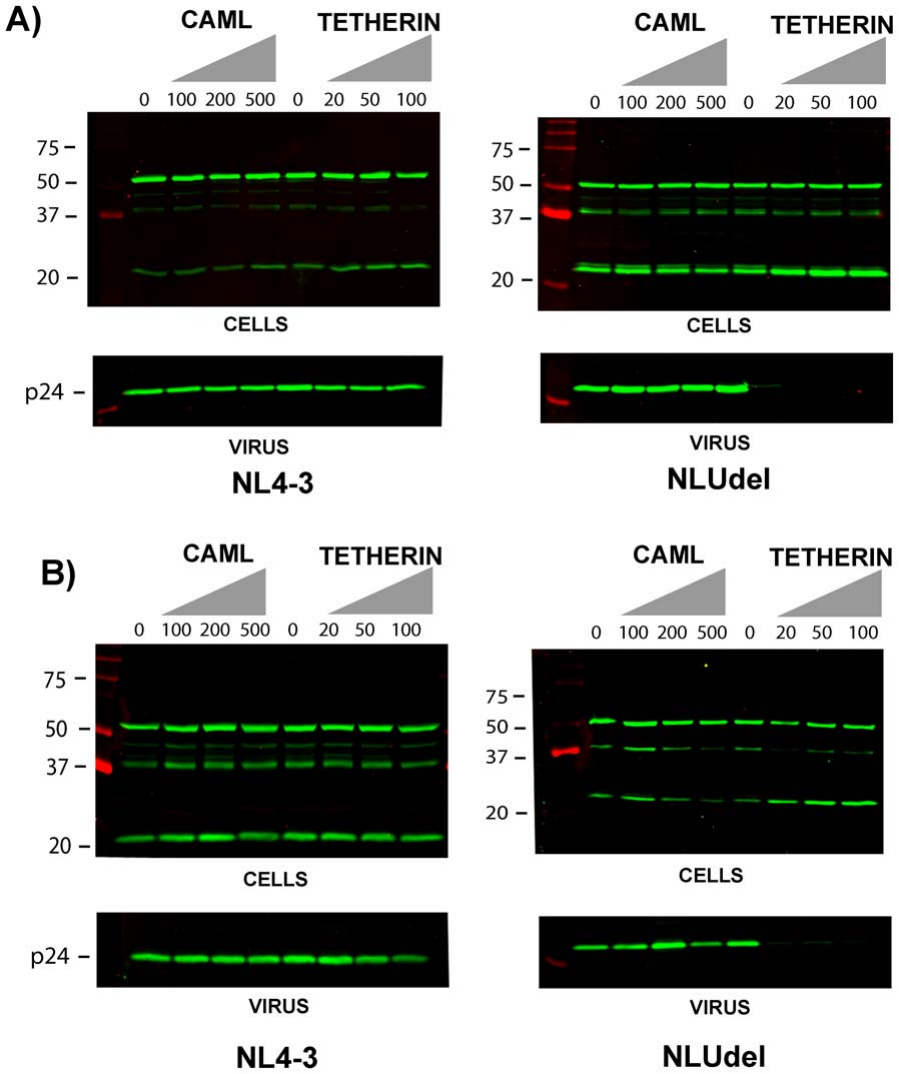
Direct comparison of CAML and tetherin effect on particle release in 293T cells and HT1080 cells. A) 293T cells were transfected with increasing amounts of CAML or tetherin expression vectors and NL4-3 (left) or NLUdel provirus (right). Cells and particles in supernatants were harvested at 48 hours post-transfection and analyzed by Western blotting using an anti-p24 monoclonal antibody. Total amount of transfected CAML or tetherin plasmid DNA per 35 mm^2^ well is indicated above the blots. B) HT1080 cells were employed in experiments identical to those described for 293T cells. No consistent effect of CAML on virus release was noted in either cell type, while tetherin potently inhibited virus release. Molecular mass markers are indicated on the left of each panel.

In order to directly compare the effects of CAML and tetherin expression in permissive human cells, we next expressed CAML or tetherin in 293T cells in a dose-escalating fashion, and compared particle output by NL4-3 and NLUdel. Using plasmid transfections ranging from 100 to 500 ng of CAML per 35 mm^2^ dish, we were unable to demonstrate restriction of NL4-3 or NLUdel (Fig. 3A, CAML lanes). In striking contrast, as little as 20 ng of tetherin expression plasmid resulted in a restriction of NLUdel, and the restriction was clearly Vpu-responsive as indicated by normal particle release by NL4-3 (Fig. 3A, tetherin lanes). These results, taken together with the depletion experiments in Fig. 2, suggest that in 293T cells CAML is unable to induce restriction, while expression of tetherin potently restricts particle release.

We reasoned that 293T cells may represent an exception to the proposed role of CAML in restricting particle release. We therefore repeated the head-to-head comparison of CAML and tetherin in HT1080 cells, a second permissive human cell line. Results were essentially identical, with no apparent restriction or influence of Vpu on particle release in cells expressing CAML (Fig. 3B, CAML lanes), while tetherin expression restricted particle release in a Vpu-dependent fashion (Fig. 3B, tetherin lanes). Thus, while CAML did not demonstrate restriction in human cells, results with tetherin agree completely with those of Neil and coworkers [13].

**Figure 4:**
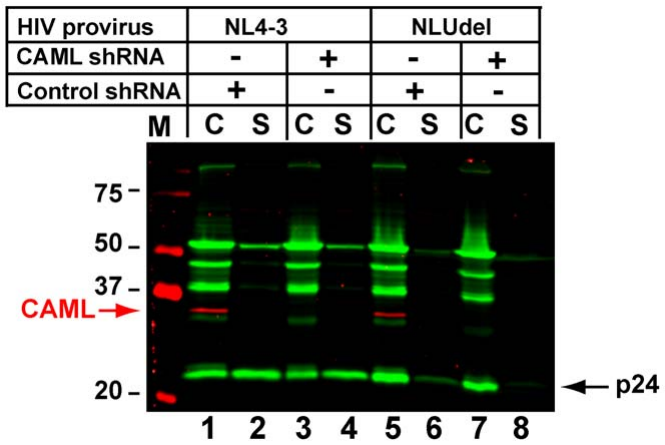
Depletion of CAML in HeLa cells fails to relieve restriction to particle release. A stably-transduced cell population demonstrating marked CAML knockdown (lanes 3, 4, 7, 8) was transfected with NL4-3 or NLUdel proviral DNA and compared with control shRNA transduced cells (lanes 1, 2, 5, 6). CAML protein is shown in red. In the presence of Vpu, particle release was equivalent in CAML knockdown or control cells (lanes 1–4). In the absence of Vpu, the restriction to particle release was present and was not affected by CAML depletion (lane 8 vs. lane 6).

The studies above in permissive cells involve expression of CAML or tetherin from expression plasmids with strong promoters, and may not reflect the endogenous restriction found in restrictive human cells. To understand the possible role that CAML plays in tetherin-mediated restriction in HeLa cells, we generated a HeLa cell line that is stably depleted of CAML. As reported previously from transient siRNA-mediated depletion [15], we expected depletion of CAML to convert these cells to the permissive, Vpu-unresponsive phenotype. However, we observed a major difference with our previous report. In Fig. 4, endogenous CAML is shown on the fluorescent blot as a red band at 34 Kd. The depletion of CAML by lentivirus-mediated shRNA expression was nearly complete by this assay (compare cell lane 3 with lane 1, and lane 7 with lane 5). Particle output by NL4-3 did not differ between control and CAML-depleted cells (supernatant lanes 2 and 4, respectively). Remarkably, depletion of CAML also failed to relieve the restriction of Vpu-deleted virus (NLUdel, supernatant lane 8). These results thus failed to support those in our previous report indicating that CAML depletion in HeLa cells relieves the restriction to particle release. These results are in complete agreement with those employing exogenous expression of tetherin in 293T cells (Fig. 2 of this report), and strongly support the ability of tetherin to restrict particle release in a manner that is independent of CAML.

**Figure 5:**
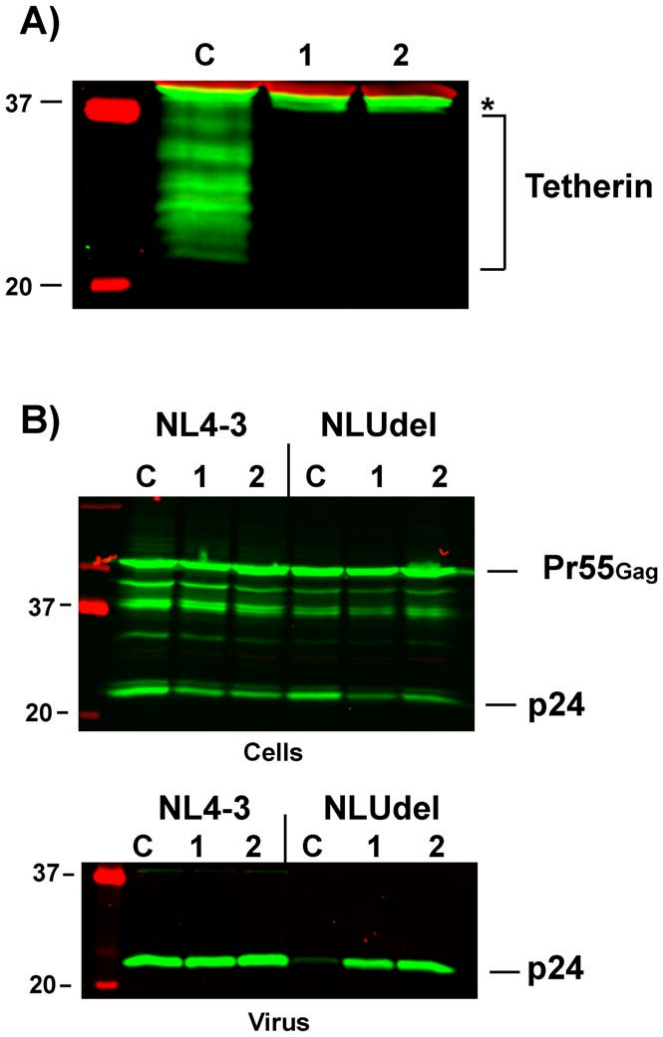
Tetherin depletion alone rescues particle release in HeLa cells. A) Two independently-derived knockdown cell populations (labeled 1,2) were employed in which knockdown of endogenous tetherin was nearly complete. Asterisk indicates a background band present in all lanes. B) Particle release defect in control shRNA-transduced HeLa cells (C) vs. knockdown cells (1, 2) is shown. Particle release was inhibited in control cells expressing NLUdel virus, while both knockdown cell populations restored particle release (NLUdel, 1 and 2 vs. C).

We next performed a similar experiment in HeLa cells that were stably depleted of tetherin. We reasoned that these cells have substantial levels of CAML (Fig. 1B), and that if CAML exhibited independent restriction of particle release that tetherin depletion alone should not relieve the restriction. Depletion of endogenous tetherin is shown for two different HeLa cell populations in Fig. 5A, compared with control shRNA transduced cells. In marked contrast to the results with CAML knockdown, depletion of tetherin relieved the restriction of particle release. Transfection of NLUdel in these tetherin-depleted cells resulted in efficient particle release (lanes 1 and 2, compare to control HeLa cells in lane C). These results are consistent with previous reports indicating that tetherin knockdown alone is sufficient to relieve the restriction to particle release. When combined with the results demonstrating substantial levels of endogenous CAML in HeLa cells (Fig. 1), these results do not substantiate an independent restrictive effect of CAML on particle release.

**Figure 6:**
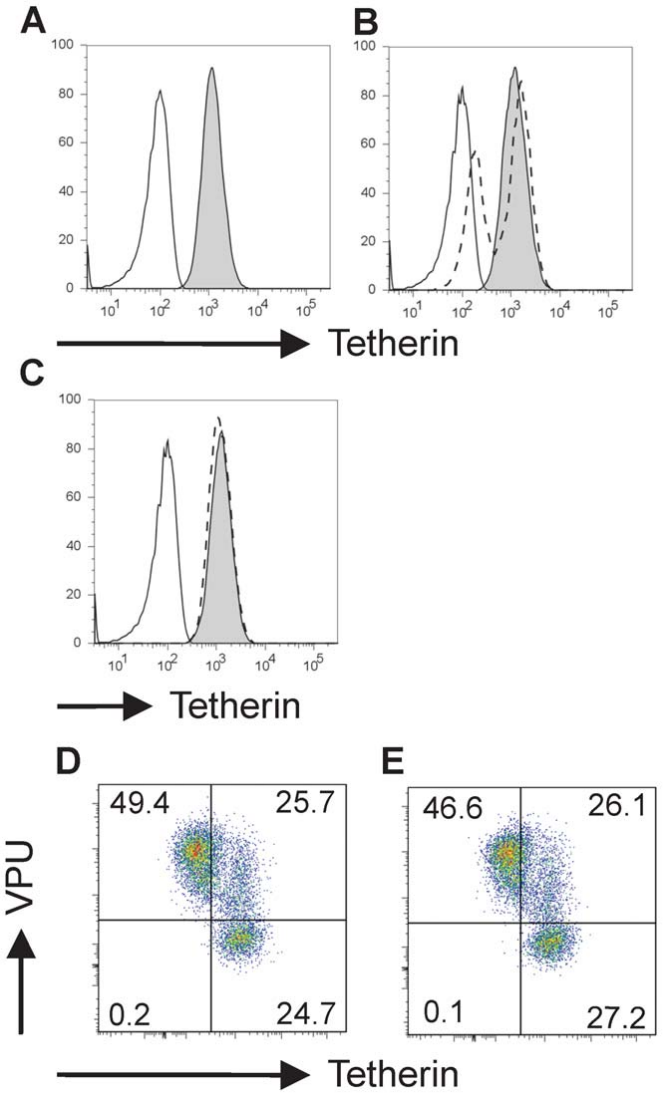
CAML does not regulate cell surface levels of tetherin. A rabbit polyclonal anti-tetherin antibody was used to quantify tetherin on the cell surface of HeLa cells by flow cytometry. A) Unstained cell control (white) and tetherin surface staining (filled histogram) using primary rabbit anti-tetherin followed by anti-rabbit APC staining. B) HeLa cells transduced with a control shRNA lentiviral vector (filled histogram) compared with unstained control cells (white). Dashed line indicates histogram of tetherin shRNA-transduced cells, demonstrating that cell surface levels were diminished by tetherin shRNA. C) Cell surface levels of tetherin in control shRNA transduced cells (filled histogram) did not differ from cells in which CAML was significantly depleted using CAML-specific shRNA (dashed lines). D) Vpu-EGFP transfected HeLa cells demonstrate cell surface downregulation of tetherin, as indicated by left shift of EGFP-positive population. E) CAML knockdown HeLa cells demonstrate cell surface downregulation of tetherin by Vpu-EGFP, similar to that shown in D.

Finally, we considered the possibility that CAML modulates cell surface levels of tetherin. Depletion of cell surface tetherin by Vpu has been reported to correlate with relief of restriction [14], although the precise role of removal of tetherin from the cell surface remains debated. We measured endogenous cell surface tetherin levels in HeLa cells in which CAML had been depleted using shRNA, and compared them to control shRNA-transduced cells. For these experiments, we utilized the HeLa cell pool that had been depleted of CAML as shown by the Western blots in Fig. 5. Unstained and surface-stained HeLa cells are shown in Fig. 6A and B, respectively. Partial tetherin depletion was employed as a control to demonstrate the shift in cell surface staining in this experiment (control shRNA shown in grey histogram, Fig. 6B vs. tetherin shRNA, dashed lines). CAML shRNA had no measurable effect on cell surface levels of tetherin, demonstrating no shift from control shRNA-transduced population (Fig. 6C, CAML-depleted cells represented by dashed lines). We then asked if the HeLa cells used in this analysis were competent for cell surface downregulation of tetherin by Vpu (expressed in these cells as an EGFP fusion protein). Vpu-EGFP efficiently downregulated tetherin from the cell surface of control cells (Fig. 6D, note shift to left upper quadrant). CAML shRNA-treated cells had no effect on this phenotype, appearing essentially identical (Fig. 6E). Together, the flow cytometry data presented here indicate that depletion of cellular CAML did not modulate cell surface tetherin, and that Vpu's ability to downmodulate cell surface tetherin was similarly unaffected by changes in cellular levels of CAML. Alltogether, the data do not support a significant role for CAML in modulating tetherin-mediated restriction of particle release; neither do they support an independent restriction of particle release by CAML.

## Discussion

Tetherin/BST-2 was identified in early 2008 as the host cell restriction that is counteracted by Vpu [13], [14]. Tetherin is an interferon-responsive gene product, which matches well the phenotype previously described by the Bieniasz laboratory [12]. The activity of tetherin in restricting retrovirus release has subsequently been extended to filoviruses [25], [26], and the impact of the depletion of tetherin by Vpu or by SIV Nef on particle release has been confirmed in a number of reports [27], [28], [29], [30]. We reported the identification of CAML in 2008 as a Vpu-interacting protein that appeared promising as an independent restriction factor or as a potential cofactor for tetherin-mediated restriction [15]. CAML demonstrated many attractive features as a potential cofactor, in particular its influence on the trafficking of transmembrane receptors such as the EGF receptor to the cell surface [23]. We postulated that recycling of tetherin to the cell surface might require CAML, providing an explanation for enhanced restriction upon the overexpression of CAML and relief of restriction upon CAML depletion [15].

Here we report that there is no influence of CAML on tetherin-mediated restriction of HIV particle release. We could not identify any effect on cell surface levels of tetherin upon depletion of CAML, nor could we demonstrate a reproducible restriction to particle release by CAML when compared directly with tetherin. During the course of the present study, it became apparent that some of the findings from our previous report indicating that CAML plays a role in restriction could not be substantiated. In particular, depletion of CAML did not relieve the restriction to particle release in HeLa cells. We cannot adequately explain this discrepancy with our previous findings, other than by implicating experimental error or misinterpretation of nonspecific toxic effects as specific restriction. We present strong arguments here, using a number of experimental approaches, that CAML is not modulating tetherin's effects or providing an independent restriction to particle release. When compared head-to-head with tetherin in human cells as we demonstrate in this report, no restriction to particle release by CAML was appreciated. In support of these data, we have received initial verification of the lack of restriction by CAML from investigators working within two other laboratories in the field to whom we sent CAML expression constructs (data not shown). A recent publication also reports the lack of effect of CAML on restriction of HIV particle release, in agreement with our recent findings [31]. These findings have led us to initiate a retraction of the previous publication reporting that CAML is a restriction factor that is overcome by Vpu, although not all authors were in agreement [15]. We anticipate that the head-to-head comparison presented here will be of some benefit in clarifying this issue, and in preventing investigators from further pursuing the relationship of CAML and tetherin. In summary, the data presented here support a model in which tetherin acts alone as a Vpu-responsive restriction factor to particle release.

## Materials and Methods

### Ethics Statement

Animals for production of antisera were housed and handled at Cocalico Biologicals, Inc., Reamstown, PA. All animals were handled in strict accordance with good animal practice in accordance with NIH's Office of Laboratory Animal Welfare as reviewed by the Institutional Animal Care and Use Committee (IACUC) at Cocalico Biologicals (Animal Welfare Assurance number A3669-01).

### Cell Lines, Plasmids and Primers

293T, HeLa, and Cos-7 cells were obtained from the American Type Culture Collection (ATCC) and were maintained in Dulbecco's Modified Eagle Medium (DMEM) supplemented with 10% fetal bovine serum (FBS) and penicillin/streptomycin. A3.01 cells (a gift from Klaus Strebel, NIH) and Jurkat cells (ATCC) were propagated in RPMI-1640 supplemented with 10% FBS, 2 mM L-glutamine, and penicillin/streptomycin. Tetherin/BST2 expression constructs were created from a cDNA clone from Origene. HA-tetherin was created by PCR amplification of the tetherin gene using primers as a Sal-Xho1 fragment and ligation of this fragment into pCMV-HA (Clontech). Primers were ACGCGTCGACCATGGCATCTACTTCGTATGAC (Sal1, forward) and CCGCTCGAGTCACTGCAGCAGAGCGCTGAG (Xho1, reverse). The full-length sequence of tetherin was confirmed by automated sequencing. CAML plasmids and Vpu expression plasmids have been previously described [15].

### Transfection and Western Blot Analysis

293T or HT1080 cells were transfected with the indicated amount of CAML or tetherin expression cDNA and pNL4-3 or NLUdel plasmids using Lipofectamine 2000 (Invitrogen). Cells and supernatants were harvested 48 hours following transfection. Particles in supernatant fractions were harvested by centrifugation at 100,000×*g* through a 20% sucrose cushion in 1.5 ml microcentrifuge tubes using an M120-SE micro-ultracentrifuge (Thermo Scientific). Cell lysates and particles were subjected to SDS-PAGE followed by Western blotting for detection of Gag proteins. Monoclonal antibody CA-183 was produced from hybridoma 183-H12-5C provided by Bruce Chesebro and Hardy Chen through the NIH AIDS Reference and Reagent Program, and was employed as the primary antibody for immunoblotting, followed by detection with anti-mouse IgG conjugated to infrared dye (IRDye 800 goat anti-mouse) and detection on the Odyssey infrared imager (Li-Cor Biosciences). In some experiments, CAML protein in cell lysates was detected using polyclonal rabbit antiserum against CAML that was produced in our laboratory. This antiserum was produced by inoculating rabbits with purified protein representing the cytoplasmic domain of CAML. To do this, the cDNA representing the cytoplasmic domain of CAML was cloned into vector pGEX-6p-1 (GE Lifesciences) and recombinant protein purified from bacteria using glutathione agarose. Purified protein cleaved from beads using Prescission protease was then purified by FPLC and utilized to inoculate rabbits in a commercial protocol carried out by Cocalico Biologicals. The specificity of the antiserum was initially verified by ELISA and Western blotting in comparison with antiserum provided by Richard Bram (Mayo Clinic, Rochester, Minnesota). For detection of CAML on Western blots, we used a secondary anti-rabbit antibody conjugated to a second infrared dye (IRDye 600 Goat anti-rabbit IgG, Li-Cor). Polyclonal rabbit antiserum directed against tetherin was produced in a similar manner. Briefly, a GST-tetherin construct in plasmid pGEX-6p-1 (GE Lifesciences) encoding the ectodomain of tetherin was employed to produce recombinant protein in bacteria. The recombinant protein was cleaved from glutathione resin using Prescission protease (GE Lifesciences) and purified by FPLC. The recombinant protein was injected into rabbits by Cocalico Biologicals, and antisera specificity verified by ELISA and Western blotting against tetherin.

### shRNA Knockdown and Preparation of Stable Knockdown Cells

Lentiviral vectors from Open Biosystems were employed to attain stable knockdown of CAML or tetherin in the indicated cell lines. Recombinant lentiviruses were produced by cotransfecting 293T cells with 5 µg lentiviral vector expressing CAML or tetherin shRNA, and 3.3 µg lentiviral packaging vectors psPAX2, and 1.25 µg vesicular stomatitis virus G glycoprotein (VSV-G) expression vector pMD2g using Lipfectamine 2000 (Invitrogen). Lentivirus stocks were harvested at 72 hours post- transfection and filtered through 0.45-µm syringe filters. A lentiviral vector containing the green fluorescent protein (GFP) expression cassette was used as a positive control for lentivirus production, and a lentiviral vector containing scrambled shRNA was used as negative control. The optimal shRNA from a series of four constructs were first identified following Western blotting, then used to generate a stably-transduced cell population. Briefly, HeLa or 293T cells were transduced with viruses in the presence of polybrene (5 µg/ml) and selected for stable integrants by culturing in complete medium containing puromycin (1 µg/ml). After 5–7 days of selection, there were no viable cells in mock wells and puromycin resistance polyclonal cell populations were isolated for further studies. Confirmation of stable knockdown was obtained by Western blotting for CAML or tetherin as already described.

### Cell Surface Staining for Tetherin

Rabbit polyclonal anti-tetherin antibody was incubated with HeLa cells, followed by staining with anti-rabbit IgG conjugated to APC (BD Biosciences). Flow cytometry was performed using a FACSCanto cytometer, and analysis performed using FlowJo software (Tree Star Inc.).
